# Test–Retest Reliability of Maximal and Rapid Knee Extensor Force Production: Influence of Verbal Instruction and Trial Selection Criteria

**DOI:** 10.1111/sms.70216

**Published:** 2026-02-10

**Authors:** Alexander Bach Sørensen, Jonas Mathiesen, Per Aagaard

**Affiliations:** ^1^ Department of Sports Science and Clinical Biomechanics, Muscle Physiology and Biomechanics Research Unit University of Southern Denmark Odense Denmark

**Keywords:** contractile impulse, explosive strength, isometric strength, knee extensor, rate of torque development, reliability, verbal instruction

## Abstract

Reliable assessments of maximal and rapid muscle strength are essential for evaluating neuromuscular performance and adaptations. This study examined the effects of verbal instruction and trial selection criteria on performance outcomes and intersession test–retest reliability during maximal isometric knee extensor testing. Twenty‐three physically active, healthy adults completed three separate test sessions, performing isometric knee extensor contractions under three standardized verbal instructions: Hard and Fast (HF; “as hard and fast as possible”), Fast Only (FO; “as fast as possible”), and Hard Only (HO; “Gradual increase in force”). Rate of torque development (RTD) and impulse were calculated using three selection criteria: the trial with the highest peak torque (Max_Peak_Torque), the trial with the highest 200‐ms impulse (Max_Impulse200), and the highest value for each time interval (Max_Composite_Interval). Both FO and HF instructions demonstrated good‐to‐excellent reliability, reflected by intraclass correlation coefficients (ICC) and within‐subject coefficients of variation (CV_w‐s_) for RTD and impulse (ICC = 0.75–0.93, CV_w‐s_ = 8%–15%), with improved reproducibility between sessions 2–3. Selection criteria strongly influenced test–retest reliability, with Max_Impulse200 and Max_Composite_Interval yielding higher ICCs and lower CV_w‐s_ compared with Max_Peak_Torque. FO instruction consistently produced higher RTD and impulse values than HF (*p* ≤ 0.05). Peak torque increased across sessions, yet reliability remained excellent for both HF and HO (ICC > 0.90, CV_w‐s_ < 7%). These findings indicate Fast Only instruction and selection of the Max_Impulse200 selection criterion may enhance reproducibility and magnitude of rapid knee extensor force assessments, especially after familiarization.

## 1 Introduction

Reliable assessments of muscle strength and rapid force capacity are essential for accurately evaluating training adaptations, functional decline, or clinical recovery. High test–retest reliability is critical to ensure that observed changes reflect true physiological adaptations rather than measurement variability, particularly when assessing training‐induced or longitudinal changes where poor reliability may mask meaningful changes. Knee extensor strength is frequently examined because it underpins daily activities and athletic performance. Isometric knee extensor peak torque has shown excellent intersession test–retest reliability (ICC > 0.90) across diverse populations [1, 2, 3], whereas measures of rapid torque development (RTD) appear to be more variable, particularly during the early phase of rising muscle force (< 100 ms) [4, 5, 6].

This variability observed across repeated measurements of peak torque and RTD may be attributable to the specific set of verbal instructions provided, while also potentially being affected by the specific trial selection criteria employed during subsequent data analysis. During lower limb strength testing, participants are typically instructed to contract the knee extensors “as hard and fast as possible” (HF; Hard and Fast), allowing simultaneous assessment of peak torque and rapid force capacity (RTD, impulse) [1, 5, 7, 8, 9]. Alternative sets of instructions include contracting “as fast as possible” (FO; Fast Only), which emphasize rapid torque generation over maximal peak torque [10, 11, 12], or “as hard as possible” with a gradual build‐up of force (HO; Hard Only), prioritizing maximal torque generation without explosive intent [13, 14]. The differential influence of verbal instructions is not trivial, as this can substantially affect performance during strength testing [10, 11, 12, 13, 14, 15].

Different trial selection criteria have been used in the literature, with the trial that produced the highest peak torque (Max_Peak_Torque) commonly selected for analysis of rapid force capacity [5, 7, 8, 9, 16]. More recent studies have opted to select the trial with the highest impulse from the onset of force (*t* = 0) to 200 ms $\left(\int_{0}^{200}\text{Torque}\;\mathrm{dt}\right)$(Max_Impulse200) for the analysis of RTD/impulse [4, 17]. However, neither of these selection criteria may necessarily produce a measure of true maximal RTD, especially not in the early phase of rising muscle force (< 100 ms). Peak torque occurs late in the contraction (typically > 300 ms from onset of contraction [7]) and is therefore weakly coupled to early RTD, while impulse over 0–200 ms represents the accumulated torque over time, where the contribution from the very initial phase (e.g., 0–30 or 0–50 ms) is small compared to later time intervals (e.g., 0–100 or 0–200 ms), which are greater in both duration and torque magnitude. Because high peak torque or impulse200 values may not necessarily coincide with maximal RTD or impulse values in the early time intervals of rising muscle force (e.g., 0–30 and 0–50 ms), selecting a single trial based on global (i.e., highest peak torque) or late‐phase (i.e., impulse 200 ms) performance criteria may increase between‐trial variability in early‐phase outcomes, thereby potentially reducing test–retest reliability. Therefore, the present study also examined test–retest reliability using a composite selection criterion based on the highest individual knee extensor RTD and impulse values for each time interval (Max_Composite_Interval). To the authors' best knowledge, this approach has not been systematically investigated in previous studies.

The present study aimed to assess the test–retest reliability of isometric knee extensor peak torque, RTD and contractile impulse over three separate test sessions separated by approximately 5–7 days in physically active healthy young adults. Specifically, we examined the impact of different verbal instructions (HF vs. FO and HF vs. HO) and selection criteria (Max_Peak_Torque, Max_Impulse200, Max_Composite_Interval) on test performance and reliability estimates. It was hypothesized that the composite time criteria (Max_Composite_Interval) would result in better reliability estimates compared to more global selection criteria (Max_Peak_Torque and Max_Impulse200).

## 2 Material and Methods

### 2.1 Participants

This study was conducted at the University of Southern Denmark from September 2024 to February 2025. The study enrolled male and female participants younger than 30 years. Exclusion criteria included participation in more than two weekly resistance or endurance training sessions targeting the lower limbs. Additionally, participants with pain or injuries of the lower extremities were deemed ineligible.

Recruitment occurred through social media and posters displayed at The University of Southern Denmark and UCL University College Lillebaelt from October 2024 to February 2025. A total of 30 participants showed interest. Based on convenience sampling, a total of 23 individuals were included. All participants received detailed information about the study's purpose and any potential risks associated with participation. Written and oral informed consent was subsequently obtained prior to inclusion in the study. The study protocol was deemed not notifiable for ethical approval by The Regional Committees on Health Research Ethics for Southern Denmark (S‐20232000‐150). Participants had a mean age of 24.1 (2.3) years, body mass: 78.5 (12.5) kg, fat free mass: 62.4 (12.0) kg, fat mass: 16.2 (4.8) kg, body mass index: 24.7 (3.2) kg·m^−2^ without differences between the groups defined by order of testing (see below) (*p* ≥ 0.530 for all comparisons). Data are presented as means and standard deviations (SD).

### 2.2 Study Overview

**FIGURE 1:**
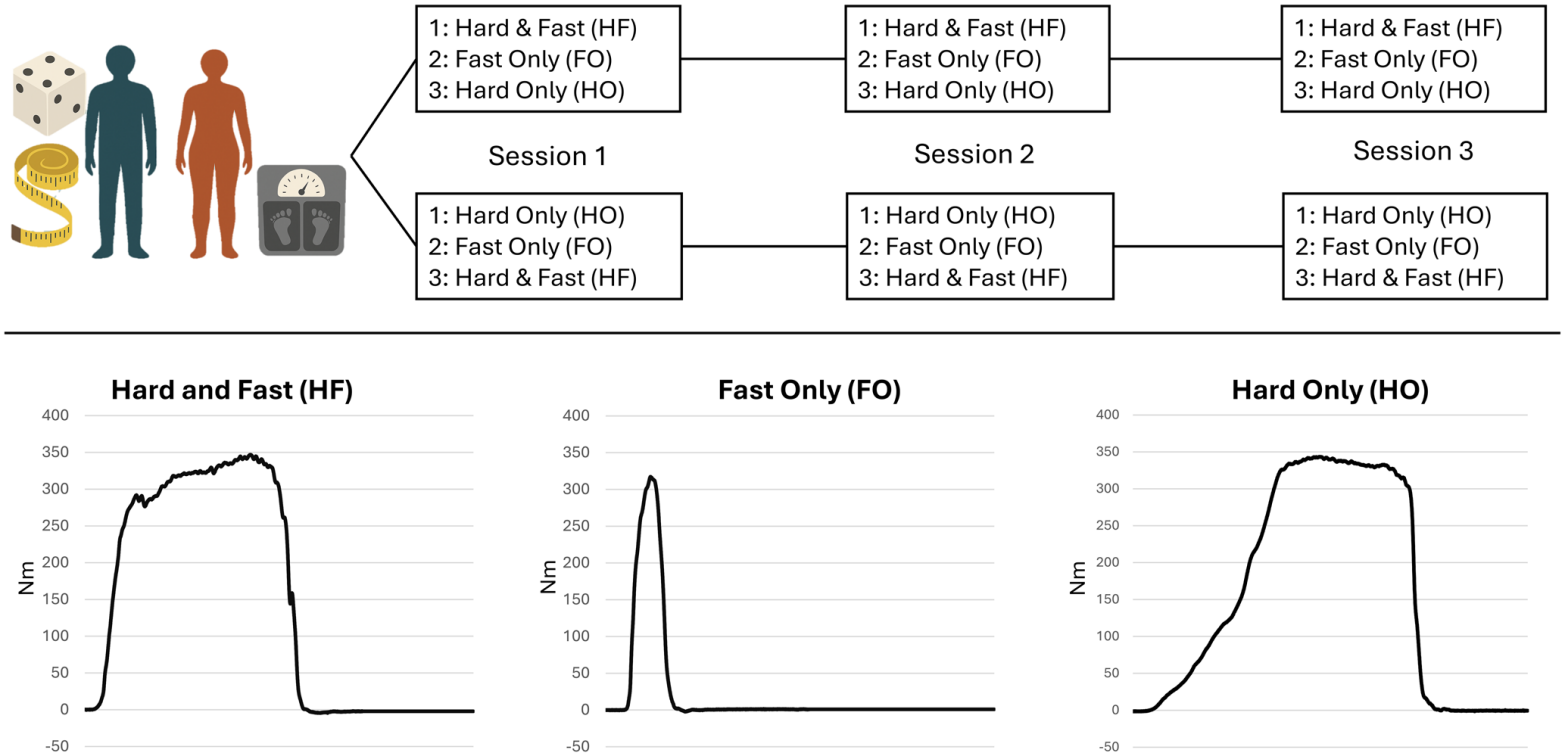
Study overview. Anthropometric and body composition data were collected in session 1. Following randomization to either HF‐FO‐HO or HO‐FO‐HF order, participants completed isometric knee extensor tests. Example torque‐time curves from one participant illustrate Hard and Fast (HF), Fast Only (FO), and Hard Only (HO) contractions.

Participants visited our laboratory three times over a maximum of 25 days, with 5.8 ± 4.4 (±SD) days between successive test sessions. Following inclusion, participants were randomly assigned to receiving either HF‐FO‐HO or HO‐FO‐HF, which represented the order of testing (Figure 1). Randomization to the different test sequences was stratified in relation to gender (gender: male/female) and training status (weekly training: yes/no). In all test sessions, participants performed isometric knee extensor muscle testing while receiving three sets of verbal instructions in the order of instruction corresponding to their group (HF‐FO‐HO or HO‐FO‐HF).

Testing was performed by the same test leader (ABS) and at the same time of day (within 1.30 ± 1.42 h) to minimize the influence of inter‐rater variance and diurnal effects. In the first test session, body height, body mass, and body composition were measured with a stadiometer and bioelectrical impedance device (Tanita MC‐780).

Participants were instructed not to change their dietary or physical activity habits while involved in the study. On testing days, they abstained from substances known to acutely affect performance (e.g., caffeine, beta‐alanine) and avoided strenuous exercise for at least 48 h beforehand.

### 2.3 Verbal Instructions

Three standardized sets of instructions were examined for their effects on peak torque and rapid force production (RTD, Peak RTD, impulse). In addition, the between‐session test–retest reliability of these three sets of instructions was examined as well. Specifically, HF and FO instructions were applied for peak RTD and RTD/impulse across fixed intervals (0–30, 0–50, 0–100, 0–200 ms), while HF and HO instructions were employed for the assessment of peak torque.

#### 2.3.1 Hard and Fast (HF) Instruction

During the HF instruction, participants were instructed to “Push as hard and fast as possible and to continue the contraction as long as the test leader shouted: PUSH, PUSH, PUSH!”. With this instruction, participants performed five trials (~3–5 s duration each) separated by two minutes of rest. If the fifth trial produced the highest peak torque, additional trials were performed until no further improvement occurred.

#### 2.3.2 Fast Only (FO) Instruction

During the Fast Only instruction (FO), participants were instructed to “Push as fast as possible, with no concern about reaching maximal force production”. To avoid ballistic muscle contractions reaching only small (yet very explosive) force amplitudes, participants were required to reach at least 70% of the highest torque from the first instruction of the day (HF or HO) [18, 19]. For trials below this threshold, participants were instructed to “Push a bit harder but keep the focus on pushing as explosively and fast as possible with no concern about reaching maximal force production”. Ten trials reaching torque values above 70% were performed interspaced by 30 s of rest.

#### 2.3.3 Hard Only (HO) Instruction

During the Hard Only instruction (HO), participants were instructed to “Gradually increase force production to roughly reach maximal force production after two seconds and to continue pushing with maximal effort for two seconds”. Following the initial two seconds, the test leader signaled maximal effort by shouting “PUSH, PUSH, PUSH!” until a clear decline in torque was observed. Participants performed 5 trials separated by 2 min of rest, with additional trials being performed if the fifth trial resulted in the highest peak torque production across all trials.

Strong verbal encouragement and online visual feedback were provided to the participants during and after all contractions. In each trial, contractions were initiated using a standardized verbal countdown (“3‐2‐1‐PUSH”) provided by the test leader. Between each set of instructions, participants rested for 5 min and were allowed to leave the test dynamometer. Test session 1 lasted approximately an hour, while test sessions 2 and 3 lasted about 45 min each.

### 2.4 Selection Criteria

For the test–retest reliability measures obtained for peak torque, the Max_Peak_Torque criterion was always used (i.e., selecting the trial with the highest peak torque measured in each test session). For the rapid force outcomes RTD, peak RTD, and impulse, three selection criteria were compared: (i) Max_Impulse200 defined as selecting the trial with the highest impulse in the first 0–200 ms of force development, and from this single trial, all RTD and impulse values were extracted for all time intervals. (ii) Max_Composite_Interval in which the highest individual RTD and impulse values were identified separately for each time interval during a given test session, irrespective of whether these values originated from the same trial or not. (iii) Max_Peak_Torque involved selecting the trial with the highest peak torque, and from this trial RTD and impulse values were extracted for all time intervals.

For all HF trials, the selection criteria of Max_Impulse200, Max_Composite_Interval, and Max_Peak_Torque were compared (Tables 1, 2, 3, 6). For all FO trials, only the selection criteria Max_Impulse200 and Max_Composite_Interval (Tables 4 and 5) were compared. For HO, only the selection criteria Max_Peak_Torque were used (Table 6). Following the determination of the most reliable measures of RTD/impulse, the influence of verbal instruction on maximal and rapid force production was investigated (Table 7).

**TABLE 1:** Rapid force production for Hard and Fast (HF) instruction using Max_Impulse200 selection criterion.

Main effect of test session					Between test session reliability						
	1	2	3	*p*		ICC_1,1_	CV_w‐s_	SEM	SDD	MAE	MAPE
RTD 30, Nm·s^−1^	1257 (605)	1219 (530)	1187 (554)	0.696	1–2	0.76 [0.56; 0.89]	21.9	274	760	311	19.5
					2–3	0.75 [0.54; 0.89]	22.0	267	740	255	20.7
RTD 50, Nm·s^−1^	1561 (668)	1532 (623)	1488 (615)	0.718	1–2	0.77 [0.57; 0.90]	19.4	304	841	326	14.6
					2–3	0.80 [0.61; 0.91]	18.0	275	763	247	15.6
RTD 100, Nm·s^−1^	1473 (487)	1442 (428)	1453 (438)	0.876	1–2	0.75 [0.53; 0.88]	15.5	229	634	243	10.3
					2–3	0.91 [0.80; 0.96]	8.98	131	364	144	10.7
RTD 200, Nm·s^−1^	1062 (310)	1048 (298)	1107 (290)	0.216	1–2	0.78 [0.59; 0.90]	13.1	140	387	155	11.8
					2–3	0.88 [0.75; 0.95]	9.4	102	282	115	12.1
Peak RTD, Nm·s^−1^	1951 (693)	1925 (683)	1887 (675)	0.784	1–2	0.80 [0.62; 0.91]	15.5	303	840	337	12.2
					2–3	0.84 [0.68; 0.93]	14.0	270	749	249	12.6
Impulse 30, Nm·s	0.778 (0.27)	0.770 (0.22)	0.791 (0.26)	0.816	1–2	0.77 [0.56; 0.89]	15.1	0.118	0.328	0.14	12.7
					2–3	0.84 [0.69; 0.93]	12.2	0.097	0.268	0.10	12.7
Impulse 50, Nm·s	2.140 (0.81)	2.114 (0.71)	2.122 (0.76)	0.963	1–2	0.78 [0.58; 0.90]	16.6	0.357	0.991	0.41	12.8
					2–3	0.85 [0.71; 0.93]	13.0	0.278	0.771	0.28	13.2
Impulse 100, Nm·s	8.570 (3.02)	8.431 (2.67)	8.414 (4.12)	0.892	1–2	0.79 [0.59; 0.90]	15.2	1.306	3.620	1.46	9.74
					2–3	0.89 [0.78; 0.95]	10.3	0.879	2.437	0.88	10.4
Impulse 200, Nm·s	17.34 (5.65)	17.06 (5.10)	17.32 (5.18)	0.885	1–2	0.78 [0.58; 0.90]	14.3	2.495	6.916	2.78	8.83
					2–3	0.92 [0.82; 0.96]	8.47	1.472	4.080	1.49	9.30

*Note:* Group mean (SD).

Abbreviations: CV_w‐s_, within‐subject coefficient of variation; ICC (1,1), intraclass correlation coefficient; Impulse, contractile impulse (Nm·s); MAE, mean absolute error; MAPE, mean absolute percentage error; *p*, *p* value of main effect of test session; RTD, rate of torque development (Nm·s^−1^); SDD, smallest detectable difference; SEM, standard error of measurement.

### 2.5 Data Assessments

Warm‐up was performed on a stationary bike ergometer prior to knee extensor testing. Participants pedaled for 3 min at 1.5 W·kg^−1^, followed by three 5‐s sprints at 85% effort, each separated by 25 s of light pedaling, concluding with 30 s easy‐effort pedaling. All testing was performed using an isokinetic dynamometer (Kinetic Communicator 500H; Chattecx Corp., Hixon, TN). The right knee extensor was tested isometrically, and RTD was calculated as the average slope of the torque‐time curve from onset of contraction (2.5% of each trial's peak torque) to 30, 50, 100 and 200 ms into the phase of rising muscle force (RTD30, −50, −100 and −200) [7, 8, 9]. Likewise, contractile impulse was calculated as the area under the torque‐time curve $\left(\int \text{Torque}\;\mathrm{dt}\right)$ in the same time intervals (Impulse30, ‐50, ‐100 and ‐200) [7]. Peak RTD was calculated as the highest rate of torque development in a continuous 10‐ms moving window.

Care was taken to align the rotational axis of the dynamometer lever arm with the rotational axis of the knee joint. Participants were then fastened at the hip, distal thigh, and ankle with the knee fixed at 70°. The dynamometer load cell was positioned 2 cm above the lateral malleolus. Seat positions and lever arm length were noted at test session 1 to ensure identical test position in subsequent test sessions and to allow calculation of knee extensor torque (Nm) by multiplying the measured force (N) by the lever arm length (m).

All trials were gravity‐corrected to account for the passive weight of the lower limb [20]. Trials with visible countermovement were discarded on site and a new trial was provided. Dynamometer force signals were sampled at 1000 Hz using a 16‐bit A/D converter. During later offline analysis, all force signals were low‐pass filtered with a 4th order zero‐lag Butterworth filter with a 15 Hz cutoff frequency [8].

### 2.6 Statistical Analysis

Following tests for normality, anthropometrics were compared between groups (Order of testing) using unpaired *t*‐tests. The normality of linear mixed model (LMM) standardized residuals was tested through visual inspection of homoscedasticity, PP‐plots and Shapiro–Wilk testing, and all assumptions were met in all cases. Intersession test–retest reliability (Tables 1, 2, 3, 4, 5, 6) was assessed using the absolute agreement, single rater intraclass correlation coefficient (ICC_1,1_) [21] and was calculated from LMMs including subject ID as a random intercept and interpreted as follows: < 0.5 (Poor), 0.5–0.75 (Moderate), 0.75–0.9 (Good) and > 0.9 (Excellent) [22]. Within‐subject coefficient of variation (CV_w‐s_) [23], standard error of measurement (SEM; mean within‐subject SD), smallest detectable difference (SDD; 1.96 × √2 × SEM), mean absolute error (MAE) and mean absolute percentage error (MAPE) were further calculated. Reliability metrics were calculated across sessions 1–2 and 2–3. Systematic changes between sessions (i.e., learning effects) were investigated using LMM with test session as a fixed effect and a random intercept for subject ID, where the overall effect of test session was tested using Wald *χ* ^2^ tests. Pairwise differences between sessions (1–2, 2–3, and 1–3) were evaluated using Wald tests of pairwise contrasts obtained from the estimated marginal means. Standardized effect sizes were calculated as Cohen's *d* for pairwise contrasts obtained from the LMM, and interpreted according to [24] with 0.2, 0.5, 0.8, representing small, medium and large effect sizes, respectively. Between‐instruction differences were investigated with instruction type (HF vs. HO for Peak torque; HF vs. FO for remaining measures) and test session as fixed effects (Table 7). Additionally, instruction type‐test session interaction effects were investigated.

Data are reported as mean ± SD, while square brackets listed for ICC_1,1_, mean differences and effect sizes indicate 95% confidence intervals. All analyses were performed in Stata 19 (StataCorp).

## 3 Results

Of 30 potential participants showing interest, 23 participants were included and completed the three test sessions without injuries or protocol deviations, yielding 100% compliance. Reasons for exclusion were patella tendinopathy (*n* = 1), too high training frequency (resistance/endurance, *n* = 4/2).

Maximum FO knee extension torque with FO instruction corresponded to 83.2%–89.5% of the highest individual peak torque recorded across all verbal instructions involving maximal knee extensor torque production (HF, HO).

### 3.1 Test–Retest Reliability Across Sessions

Numerically improved test–retest reliability was observed for RTD and impulse between sessions 2–3 compared with sessions 1–2 (Tables 1, 2, 3, 4, 5). For example, with HF instruction and Max_Impulse200 or Max_Composite_Interval, ICC values for RTD100 increased from 0.75 (session 1–2) to 0.91 (session 2–3), while CV_w‐s_ decreased from 15.5% to 8.98% (Tables 1 and 2). Similar patterns were observed across rapid force measurements, indicating enhanced measurement stability following familiarization (Tables 1, 2, 3, 4, 5). Interestingly, the instruction‐dependent difference in reliability was more pronounced in non‐familiarized (test sessions 1–2) compared to familiarized conditions (test sessions 2–3). In other words, FO appeared highly reliable across all test sessions (Tables 4 and 5), whereas HF reliability was found to improve in numerical terms after the familiarization session (Session 1) (Tables 1, 2, 3).

**TABLE 2:** Rapid force production for Hard and Fast (HF) instruction using Max_Composite_Interval selection criterion.

Main effect of test session					Between test session reliability						
	1	2	3	*p*		ICC_1,1_	CV_w‐s_	SEM	SDD	MAE	MAPE
RTD 30, Nm·s^−1^	1278 (599)	1254 (555)	1199 (550)	0.592	1–2	0.80 [0.61; 0.91]	20.0	256	709	286	17.9
					2–3	0.79 [0.59: 0.90]	20.3	252	699	234	19.3
RTD 50, Nm·s^−1^	1563 (667)	1550 (637)	1495 (613)	0.725	1–2	0.79 [0.60; 0.90]	18.9	297	824	326	14.1
					2–3	0.81 [0.63; 0.91]	17.7	273	756	239	15.4
RTD 100, Nm·s^−1^	1477 (487)	1444 (421)	1453 (438)	0.856	1–2	0.75 [0.53; 0.88]	15.5	229	636	245	10.1
					2–3	0.91 [0.81; 0.96]	8.89	130	361	142	10.6
RTD 200, Nm·s^−1^	1076 (317)	1064 (295)	1112 (293)	0.341	1–2	0.80 [0.62; 0.91]	12.4	134	372	149	10.0
					2–3	0.90 [0.79; 0.96]	8.42	93.7	257	101	10.3
Peak RTD, Nm·s^−1^	1968 (694)	1931 (692)	1892 (671)	0.720	1–2	0.79 [0.60; 0.91]	15.9	313	867	349	11.9
					2–3	0.85 [0.69; 0.93]	13.7	265	735	244	12.2
Impulse 30, Nm·s	0.803 (0.26)	0.781 (0.24)	0.817 (0.31)	0.682	1–2	0.81 [0.62; 0.91]	13.6	0.109	0.302	0.12	13.1
					2–3	0.79 [0.60; 0.91]	15.5	0.126	0.349	0.11	13.9
Impulse 50, Nm·s	2.174 (0.79)	2.144 (0.74)	2.159 (0.78)	0.950	1–2	0.80 [0.62; 0.91]	15.4	0.336	0.930	0.39	10.9
					2–3	0.89 [0.77; 0.95]	11.6	0.253	0.701	0.24	11.8
Impulse 100, Nm·s	8.570 (3.02)	8.453 (2.69)	8.418 (2.74)	0.905	1–2	0.79 [0.60; 0.91]	15.0	1.291	3.578	1.43	9.69
					2–3	0.89 [0.78: 0.95]	10.3	0.878	2.434	0.87	10.4
Impulse 200, Nm·s	17.34 (5.65)	17.06 (5.10)	17.32 (5.18)	0.885	1–2	0.78 [0.58; 0.90]	14.3	2.495	6.916	2.78	8.83
					2–3	0.92 [0.82; 0.96]	8.47	1.472	4.080	1.49	9.30

*Note:* Group mean (SD).

Abbreviations: CV_w‐s_, within‐subject coefficient of variation; ICC (1,1), intraclass correlation coefficient; Impulse, contractile impulse (Nm·s); MAE, mean absolute error; MAPE, mean absolute percentage error; *p*, *p* value of main effect of test session; RTD, rate of torque development (Nm·s^−1^); SDD, smallest detectable difference; SEM, standard error of measurement.

**TABLE 3:** Rapid force production for Hard and Fast (HF) instruction using Max_Peak_Torque selection criterion.

Main effect of test session					Between test session reliability						
	1	2	3	*p*		ICC_1,1_	CV_w‐s_	SEM	SDD	MAE	MAPE
RTD 30, Nm·s^−1^	992.8 (612)	1041 (568)	954.5 (608)	0.745	1–2	0.51 [0.24;0.78]	39.6	408	1130	429	44.2
					2–3	0.81 [0.63; 0.91]	25.4	256	711	283	39.0
RTD 50, Nm·s^−1^	1235 (701)	1302 (654)	1194 (714)	0.725	1–2	0.46 [0.19; 0.76]	38.3	491	1361	542	40.3
					2–3	0.78 [0.59; 0.90]	25.0	315	874	337	45.4
RTD 100, Nm·s^−1^	1261 (515)	1310 (465)	1206 (508)	0.552	1–2	0.51 [0.24; 0.78]	26.1	340	942	369	26.8
					2–3	0.68 [0.44; 0.85]	21.5	274	759	287	34.3
RTD 200, Nm·s^−1^	1000 (308)	995 (320)	1007 (319)	0.970	1–2	0.73 [0.51; 0.88]	16.0	161	447	187	16.7
					2–3	0.76 [0.56; 0.89]	15.1	153	425	153	23.4
Peak RTD, Nm·s^−1^	1679 (727)	1703 (712)	1578 (756)	0.615	1–2	0.54 [0.26; 0.79]	28.3	485	1343	525	26.8
					2–3	0.70 [0.46; 0.86]	24.2	401	1110	390	41.3
Impulse 30, Nm·s	0.680 (0.26)	0.704 (0.24)	0.705 (0.27)	0.799	1–2	0.60 [0.34: 0.82]	21.9	0.153	0.423	0.168	17.8
					2–3	0.79 [0.60; 0.90]	16.0	0.114	0.316	0.116	16.3
Impulse 50, Nm·s	1.794 (0.80)	1.873 (0.70)	1.817 (0.81)	0.853	1–2	0.54 [0.27; 0.79]	28.1	0.521	1.443	0.578	23.1
					2–3	0.81 [0.63; 0.91]	18.1	0.337	0.935	0.361	21.9
Impulse 100, Nm·s	7.177 (3.12)	7.480 (2.83)	7.046 (3.13)	0.743	1–2	0.51 [0.23; 0.78]	27.9	2.071	5.740	2.289	25.0
					2–3	0.75 [0.54; 0.89]	20.1	1.474	4.087	1.547	28.4
Impulse 200, Nm·s	14.99 (5.76)	15.52 (5.42)	14.79 (5.92)	0.769	1–2	0.56 [0.29; 0.80]	23.8	3.672	10.18	4.018	22.6
					2–3	0.74 [0.52; 0.88]	18.8	2.886	7.999	3.031	28.0

*Note:* Group mean (SD).

Abbreviations: CV_w‐s_, within‐subject coefficient of variation; ICC (1,1), intraclass correlation coefficient; Impulse, Contractile impulse (Nm·s); MAE, mean absolute error; MAPE, mean absolute percentage error; *p*, *p* value of main effect of test session; RTD, rate of torque development (Nm·s^−1^); SDD, smallest detectable difference; SEM, standard error of measurement.

### 3.2 Effects of Selection Criteria

Trial selection exerted a substantial influence on the reproducibility of rapid force production. Using Max_Peak_Torque led to lower ICCs and higher CV_w‐s_, particularly for early RTD intervals (≤ 100 ms) (Table 3) compared to both other selection criteria (Tables 1 and 2). Both Max_Composite_Interval and Max_Impulse200 substantially improved reliability (ICC 0.75–0.92; CV_w‐s_ 8%–15%) compared to Max_Peak_Torque across all sessions (Tables 1, 2, 3), while the differences between the two optimized criteria were numerically small and not practically meaningful.

To avoid combining values from different trials (discussed later), the influence of verbal instruction (HF vs. FO) on rapid force production was investigated with the Max_Impulse200 selection criterion (Table 7). Comparisons between HF and HO regarding peak torque were performed using the Max_Peak_Torque selection criterion (Table 7).

### 3.3 Effects of Instruction

The type of verbal instruction was found to significantly influence both the performance magnitude (Table 7) and the degree of reliability (Tables 1, 2, 4 and 5). Pairwise comparisons between instructions were performed within each test session; therefore, significant differences within specific sessions may occur despite nonsignificant overall main effects of instruction, reflecting instruction‐dependent differences across sessions rather than uniform effects across time. Specifically, FO produced higher values for RTD and impulse, with significant main effects observed for RTD100 (*p* = 0.013) and Peak RTD (*p* = 0.040) (Table 7). In session 1, FO elicited greater RTD100 and Peak RTD compared with HF, whereas in sessions 2 and 3, differences extended to nearly all RTD intervals except RTD30 in session 2 (Table 7). For impulse, no main effect of instruction was detected overall, but FO produced greater impulse100 and impulse200 in session 2 (*p* = 0.011, *p* = 0.003) and higher impulse50‐200 in session 3 (all *p* ≤ 0.005) (Table 7).

**TABLE 4:** Rapid force production for Fast Only (FO) instruction using Max_Impulse200 selection criterion.

Main effect of test session					Between test session reliability						
	1	2	3	*p*		ICC_1,1_	CV_w‐s_	SEM	SDD	MAE	MAPE
RTD 30, Nm·s^−1^	1368 (630)	1374 (519)	1475 (620)	0.184	1–2	0.83 [0.67; 0.93]	16.8	233	646	259	22.1
					2–3	0.83 [0.67; 0.92]	16.1	232	643	271	21.1
RTD 50, Nm·s^−1^	1690 (658)^a^	1725 (613)^ab^	1852 (669)^b^	**0.036**	1–2	0.86 [0.72; 0.94]	13.5	234	648	255	15.9
					2–3	0.91 [0.81; 0.96]	10.7	193	536	228	14.6
RTD 100, Nm·s^−1^	1603 (466)	1611 (459)	1694 (450)	**0.047**	1–2	0.91 [0.82; 0.96]	8.31	135	374	143	9.42
					2–3	0.91 [0.82; 0.96]	8.01	134	371	132	9.47
RTD 200, Nm·s^−1^	1131 (341)^a^	1143 (315)^ab^	1223 (313)^b^	**0.013**	1–2	0.86 [0.72; 0.94]	10.5	120	333	143	13.9
					2–3	0.82 [0.65; 0.92]	11.1	133	369	147	14.2
Peak RTD, Nm·s^−1^	2122 (576)^a^	2142 (734)^ab^	2306 (758)^b^	**0.021**	1–2	0.83 [0.66; 0.92]	12.6	271	751	290	10.9
					2–3	0.93 [0.85; 0.97]	8.83	199	551	213	11.2
Impulse 30, Nm·s	0.786 (0.27)	0.800 (0.22)	0.837 (0.27)	0.287	1–2	0.71 [0.47; 0.87]	16.6	0.133	0.369	0.15	15.2
					2–3	0.82 [0.65; 0.92]	12.6	0.104	0.289	0.11	14.7
Impulse 50, Nm·s	2.226 (0.81)	2.273 (0.69)	2.404 (0.83)	0.105	1–2	0.80 [0.62; 0.91]	14.6	0.332	0.919	0.37	15.1
					2–3	0.87 [0.74; 0.94]	11.5	0.272	0.754	0.32	14.7
Impulse 100, Nm·s	9.117 (2.87)^a^	9.252 (2.69)^ab^	9.771 (2.88)^b^	**0.021**	1–2	0.90 [0.80; 0.96]	9.21	0.855	2.370	0.91	10.5
					2–3	0.93 [0.85; 0.97]	7.53	0.724	2.008	0.85	10.1
Impulse 200, Nm·s	18.46 (5.47)^a^	18.79 (5.33)^a^	19.89 (5.45)^b^	**0.004**	1–2	0.92 [0.83; 0.96]	8.16	1.537	4.261	1.59	9.31
					2–3	0.93 [0.85; 0.97]	7.36	1.439	3.988	1.52	9.04

*Note:* Group mean (SD). Mean values followed by different letters are statistically different within each RTD and impulse time interval. *p* values for significant main effects of test sessions are marked in bold.

Abbreviations: CV_w‐s_, within‐subject coefficient of variation; ICC (1,1), intraclass correlation coefficient; Impulse, contractile impulse (Nm·s); MAE, mean absolute error; MAPE, mean absolute percentage error; *p*, *p* value of main effect of test session; RTD, rate of torque development (Nm·s^−1^); SDD, smallest detectable difference; SEM, standard error of measurement.

**TABLE 5:** Rapid force production for Fast Only (FO) instruction using Max_Composite_Interval selection criterion.

Main effect of test session					Between test session reliability						
	1	2	3	*p*		ICC_1,1_	CV_w‐s_	SEM	SDD	MAE	MAPE
RTD 30, Nm·s^−1^	1381 (624)	1416 (520)	1512 (606)	0.079	1–2	0.85 [0.69; 0.93]	15.7	222	617	258	17.8
					2–3	0.87 [0.73; 0.94]	13.9	205	569	233	16.6
RTD 50, Nm·s^−1^	1702 (648)^a^	1750 (612)^ab^	1869 (662)^b^	**0.021**	1–2	0.88 [0.75; 0.95]	12.4	217	602	244	14.3
					2–3	0.92 [0.83; 0.96]	9.70	177	492	209	13.2
RTD 100, Nm·s^−1^	1603 (465)^a^	1625 (452)^ab^	1703 (453)^b^	**0.034**	1–2	0.90 [0.80; 0.96]	8.63	141	390	151	8.81
					2–3	0.93 [0.85; 0.97]	7.06	119	329	127	8.67
RTD 200, Nm·s^−1^	1171 (337)^a^	1194 (338)^ab^	1240 (324)^b^	**0.043**	1–2	0.92 [0.83; 0.96]	8.01	95.8	266	105	11.2
					2–3	0.89 [0.78; 0.95]	8.79	108	300	122	11.0
Peak RTD, Nm·s^−1^	2167 (609)^a^	2205 (752)^ab^	2334 (768)^b^	**0.028**	1–2	0.86 [0.72; 0.94]	11.3	251	695	280	8.59
					2–3	0.96 [0.91; 0.98]	6.73	154	428	174	8.60
Impulse 30, Nm·s	0.808 (0.26)	0.819 (0.22)	0.848 (0.27)	0.380	1–2	0.74 [0.52; 0.87]	14.8	0.121	0.338	0.13	12.4
					2–3	0.86 [0.72; 0.94]	10.6	0.090	0.249	0.09	11.5
Impulse 50, Nm·s	2.245 (0.80)	2.309 (0.69)	2.429 (0.81)	0.073	1–2	0.81 [0.64; 0.92]	13.8	0.317	0.879	0.36	12.8
					2–3	0.90 [0.78, 0.95]	10.1	0.241	0.668	0.28	12.4
Impulse 100, Nm·s	9.117 (2.87)^a^	9.288 (2.69)^ab^	9.784 (2.87)^b^	**0.019**	1–2	0.90 [0.80; 0.96]	9.08	0.845	2.341	0.90	9.88
					2–3	0.94 [0.86; 0.97]	7.26	0.700	1.940	0.80	9.50
Impulse 200, Nm·s	18.46 (5.47)^a^	18.80 (5.33)^a^	19.89 (5.45)^b^	**0.004**	1–2	0.92 [0.82; 0.96]	8.16	1.537	4.261	1.59	9.31
					2–3	0.93 [0.85; 0.97]	7.36	1.439	3.988	1.52	9.04

*Note:* Group mean (SD). Mean values followed by different letters are statistically different between sessions within each RTD and impulse time interval. *p* values for significant main effects of test sessions are marked in bold.

Abbreviations: CV_w‐s_, coefficient of variation; ICC (1,1), intraclass correlation coefficient; Impulse, Contractile impulse (Nm·s); MAE, mean absolute error; MAPE, mean absolute percentage error; *p*, *p* value of main effect of test session; RTD, rate of torque development (Nm·s^−1^); SDD, smallest detectable difference; SEM, standard error of measurement.

Peak torque increased significantly across sessions; however, both HF and HO instructions demonstrated excellent relative reliability (ICC > 0.90, CV_w‐s_ < 7%, MAPE < 8.4%), indicating high consistency across repeated measurements (Table 6). No differences in peak torque were observed between instructions (Table 7).

**TABLE 6:** Peak torque produced with Hard and Fast (HF) and Hard Only (HO) instructions using Max_Peak_Torque selection criterion.

Main effect of test session					Between test session reliability						
	1	2	3	*p*		ICC_1,1_	CV_w‐s_	SEM	SDD	MAE	MAPE
Peak torque—HF, Nm	312 (91)^a^	316 (82)^a^	336 (92)^b^	**< 0.001**	1–2	0.94 [0.87; 0.97]	6.6	20.78	57.6	22.3	8.36
					2–3	0.93 [0.86; 0.97]	6.3	22.35	62.0	25.6	8.36
Peak torque—HO, Nm	306 (88)^a^	319 (86)^a^	332 (89)^b^	**< 0.001**	1–2	0.95 [0.89; 0.98]	6.2	19.46	53.9	21.9	7.00
					2–3	0.95 [0.90: 0.98]	5.9	18.65	51.7	21.5	6.82

*Note:* Group mean (SD); Peak torque (Nm); mean values followed by different letters are statistically different between sessions. *p* values for significant main effects of test sessions are marked in bold.

Abbreviations: CV_w‐s_, within‐subject coefficient of variation; ICC (1,1), intraclass correlation coefficient; MAE, mean absolute error; MAPE, mean absolute percentage error; *p*, *p* value of main effect of test session; SDD, smallest detectable difference; SEM, standard error of measurement.

**TABLE 7:** Peak torque, RTD, and impulse recorded across instructions and test sessions.

	Session 1		Session 2		Session 3		Effects
	HF	HO	HF	HO	HF	HO	
Peak torque, Nm	311.6 (91)	305.8 (88)	315.6 (82)	318.9 (86)	335.7 (91)	332.3 (88)	*p*_Time_ < **0.001** *p*_Instruction_ = 0.339 *p*_Interaction_ = 0.552
	−5.88 [−17.9;6.18] *p* = 0.339; *d* = −0.28[−0.86; 0.30]		3.28 [−8.77; 15.3] *p* = 0.594; *d* = 0.16 [−0.24; 0.74]		−3.41 [−15.5; 8.64] *p* = 0.579; *d* = −0.16 [−0.74; 0.41]		

	HF	FO	HF	FO	HF	FO	
RTD30, Nm·s^−1^	1257 (605)	1369 (630)	1219 (530)	1374 (519)	1187 (554)	1476 (620)	*p*_Time_ = 0.676 *p*_Instruction_ = 0.161 *p*_Interaction_ = 0.259
	111 [−44.2;267] *p* = 0.160; *d* = 0.41[−0.16; 0.99]		155 [−0.89; 310] *p* = 0.051; *d* = 0.58 [−0.003; 1.15]		289 [133; 444] *p* < **0.001**; *d* = 1.07 [0.49; 1.65]		
RTD50, Nm·s^−1^	1561 (668)	1690 (658)	1533 (623)	1725 (613)	1488 (615)	1852 (669)	*p*_Time_ = 0.679 *p*_Instruction_ = 0.125 *p*_Interaction_ = 0.121
	129 [−35.6; 293] *p* = 0.125; *d* = 0.45 [−0.13; 1.03]		192 [27.8; 356] *p* = **0.022**; *d* = 0.68 [0.10; 1.25]		364 [200; 528] *p* < **0.001**; *d* = 1.28 [0.70;1.86]		
RTD100, Nm·s^−1^	1473 (487)	1603 (466)	1442 (428)	1611 (459)	1452 (438)	1694 (450)	*p*_Time_ = 0.838 *p*_Instruction_ = **0.013** *p*_Interaction_ = 0.311
	130 [27.0; 233] *p* = **0.013**; *d* = 0.73 [0.15; 1.31]		169 [66.1; 272] *p* = **0.001**; *d* = 0.95 [0.37: 1.53]		242 [139; 345] *p* < **0.001**; *d* = 1.36 [0.78; 1.94]		
RTD200, Nm·s^−1^	1062 (310)	1131 (315)	1048 (298)	1143 (315)	1108 (290)	1223 (313)	*p*_Time_ = 0.233 *p*_Instruction_ = 0.060 *p*_Interaction_ = 0.663
	68.9 [−2.91; 141] *p* = 0.060; *d* = 0.55 [−0.02; 1.13]		94.8 [23.0; 167] *p* = **0.010**; *d* = 0.76 [0.19; 1.34]		116 [44.0; 188] *p* = **0.002**; *d* = 0.93 [0.35; 1.51]		
Peak RTD, Nm·s^−1^	1951 (693)	2122 (576)	1925 (683)	2142 (734)	1887 (675)	2306 (758)	*p*_Time_ = 0.736 *p*_Instruction_ = **0.040** *p*_Interaction_ = 0.080
	170 [7.42; 333] *p* = **0.040**; *d* = 0.60 [0.03; 1.18]		217 [53.7; 380] *p* = **0.009**; *d* = 0.77 [0.19; 1.35]		419 [256; 582] *p* < **0.001**; *d* = 1.49 [0.91; 2.07]		
Impulse 30, Nm·s	0.778 (0.27)	0.786 (0.27)	0.770 (0.22)	0.800 (0.22)	0.791 (0.27)	0.837 (0.27)	*p*_Time_ = 0.834 *p*_Instruction_ = 0.801 *p*_Interaction_ = 0.763
	0.009 [−0.060; 0.078] *p* = 0.801; *d* = 0.07 [−0.50;0.65]		0.030 [−0.039; 0.099] *p* = 0.397; *d* = 0.25 [−0.33; 0.83]		0.046 [−0.024; 0.115] *p* = 0.197; *d* = 0.38 [−0.20;0.96]		
Impulse 50, Nm·s	2.140 (0.81)	2.226 (0.81)	2.114 (0.71)	2.273 (0.69)	2.122 (0.76)	2.404 (0.83)	*p*_Time_ = 0.963 *p*_Instruction_ = 0.391 *p*_Interaction_ = 0.370
	0.085 [−0.110; 0.280] *p* = 0.391; *d* = 0.25 [−0.33; 0.83]		0.160 [−0.036; 0.355] *p* = 0.109; *d* = 0.47 [−0.11; 1.05]		0.282 [0.087; 0.477] *p* = **0.005**; *d* = 0.84 [0.26; 1.41]		
Impulse 100, Nm·s	8.570 (3.02)	9.117 (2.87)	8.431 (2.67)	9.252 (2.69)	8.414 (2.74)	9.771 (2.88)	*p*_Time_ = 0.868 *p*_Instruction_ = 0.090 *p*_Interaction_ = 0.200
	0.547 [−0.085; 1.180] *p* = 0.090; *d* = 0.50 [−0.08; 1.08]		0.821 [0.189; 1.453] *p* = **0.011**; *d* = 0.75 [0.17;1.33]		1.357 [0.725; 1.990] *p* < **0.001**; *d* = 1.24 [0.66;1.82]		
Impulse 200, Nm·s	17.34 (5.65)	18.46 (5.47)	17.06 (5.10)	18.79 (5.33)	17.32 (5.18)	19.90 (5.45)	*p*_Time_ = 0.858 *p*_Instruction_ = 0.053 *p*_Interaction_ = 0.203
	1.116 [−0.014; 2.247] *p* = 0.053; *d* = 0.57 [−0.01; 1.15]		1.1732 [0.602; 2.863] *p* = **0.003**; *d* = 0.89 [0.30;1.64]		2.567 [1.437; 3.698] *p* < **0.001**; *d* = 1.31 [0.74; 1.89]		

*Note:* Hard and Fast (HF) and Hard Only (HO) with Max_Peak_Torque selection criteria. Hard and Fast (HF) and Fast Only (FO) with Max_Impulse200 selection criteria. Group means (SD) and between‐group differences [95% CI]; Positive differences and effect sizes favor HO and FO over HF. Significant between‐instruction differences and effects of test sessions are marked in bold.

Abbreviations: FO, Fast Only; HF, Hard and Fast; HO, Hard Only; Impulse, contractile impulse (Nm·s); *p*_Instruction_, *p* value for main effect of instruction; *p*_Interaction_, *p* value for interaction between Time and Instruction. Peak torque (Nm); *p*_Time_, *p* value for main effect of time; RTD, Rate of torque development (Nm·s^−1^).

## 4 Discussion

The present study examined the influence of different types of verbal instruction and selection criteria on strength testing performance and intersession test–retest reliability of isometric knee extensor peak torque, RTD, peak RTD and contractile impulse in physically active healthy adults across three separate test sessions. The main findings were that using a selection criterion based on highest 200 ms impulse (Max_Impulse200) or composite time interval analysis (Max_Composite_Interval) resulted in good‐to‐excellent test–retest reliability and improved reliability for all RTD and impulse variables compared to a selection criterion based on highest peak torque (Max_Peak_Torque) for the HF instruction. FO demonstrated numerically better reliability than HF for both Max_Impulse200 and Max_Composite_Interval. Increased test–retest reliability was observed for test sessions 2–3 compared to sessions 1–2, suggesting improved reliability following familiarization. Generally improved reproducibility was further observed for RTD/impulse when analyzed in longer (≥ 100 ms) time intervals compared to shorter (≤ 50 ms) for RTD/impulse across instructions and selection criteria. Excellent test–retest reliability was noted for knee extensor peak torque irrespective of verbal instruction (HF and HO) both between test sessions 1–2 and 2–3. Conversely, the type of verbal instruction had a clear effect on rapid force capacity, as FO elicited greater RTD and impulse values compared to the HF instruction, especially when participants were familiarized with the test procedures (i.e., test sessions 2 and 3). FO elicited a consistent enhancement in rapid force capacity during later test sessions, while peak torque increased for both HF and HO instructions (overall time effect).

### 4.1 Influence of Selection Criteria on Rapid Force Production

The choice of selection criterion had a strong effect on the reproducibility of the present rapid force indices. Specifically, trials selected based on Max_Impulse200 or Max_Composite_Interval produced largely equivalent and good‐to‐excellent test–retest reliability for the present RTD, Peak RTD, and impulse variables. In contrast, the peak torque criterion (Max_Peak_Torque) resulted in reduced (poor‐to‐good ICC) RTD/impulse reliability.

The combination of HF instruction and selection of the trial with highest peak torque is a common methodological approach in the assessment of RTD and impulse in both clinical and nonclinical research settings [1, 5, 7, 8, 9]. As an alternative to this approach, improvements in test–retest reliability for RTD and impulse measures were achieved when using Max_Impulse200 or the Max_Composite_Interval selection criteria as demonstrated in the present study by higher ICC values and lower CV_w‐s_, SEM, SDD, MAE, and MAPE with the two latter selection criteria. Notably, and in disfavor of our initial study hypothesis, only minimal improvements in reliability estimates were observed when the composite time interval criterion (Max_Composite_Interval) was compared to the max impulse criterion (Max_Impulse200).

The present data show that intersession test–retest reliability is strongly influenced by the selection criterion used. Yet, limited research has addressed how instructional variations affect the reliability of rapid force production. Dirnberger et al. [25] reported improved early phase knee extensor RTD test–retest reliability when selecting the trial with highest peak RTD compared to selecting the trial with highest peak torque, manifested by ICC values for RTD30 increasing from 0.62 to 0.80. In their study, the effect of selection criteria was clear at early intervals (0–30, 0–50 ms) but diminished and became nonsignificant at longer durations (0–100, 0–200, 0–300 ms) [25].

In contrast, Speedtsberg et al. [26] recently observed improved intersession test–retest reliability for knee extensor RTD obtained at 0–50 and 0–100 ms when selecting the trial with highest peak torque compared to selecting the trial with highest RTD100. In the present study, we did not assess the Max_Peak_Torque criterion for FO instruction, as participants were told to disregard maximal force output and solely “push as fast as possible”. Notably, Speedtsberg et al. [26] also found that selecting trials based on the highest RTD100 resulted in greater RTD50 values compared with those obtained using a peak torque‐based selection criterion. These contrasting data highlight the delicate balance between optimizing test–retest reliability and validity associated with a given selection criterion, since high reliability may be of little worth if it is gained at the cost of attenuated explosive strength (RFD, impulse) outputs. In line with this notion, an argument against the composite selection criteria Max_Composite_Interval could be that the assembling of RTD and impulse maxima obtained from different trials produces a synthetic trial‐profile that may never have existed during testing.

### 4.2 Rapid Force Production

FO instruction consistently produced higher rapid force outcomes than HF across test sessions (cf. Table 7). Differences were limited to RTD100 and Peak RTD in session 1 with progressively more measures demonstrating significant differences in sessions 2–3. Importantly, although no significant main effects of time were observed (cf. Table 7), increases in RTD and impulse across test sessions (i.e., learning effects) were evident specifically for the FO condition when examined separately (cf. Tables 4 and 5), while absent in HF (cf. Tables 1 and 2), indicating an instruction‐specific learning effect.

The present observation of superior rapid force production while utilizing a solely explosive intent aligns with previous observations showing that a specific focus on explosiveness enhances measures of rapid force production relative to dual‐focus instructions [10, 11, 13, 15, 27, 28]. However, divergent findings also exist when comparing HF and FO instructions in time‐specific intervals (e.g., 0–50, 0–100, 0–200 ms) [12, 29]. Specifically, McCormick et al. [29] performed isometric mid‐thigh pull testing and found no significant difference between HF and FO instructions for early and late phase impulse. In contrast, Kozinc et al. [12] demonstrated greater rapid force capacity for ballistic (FO) compared to sustained (HF) contractions, for late‐phase knee extensor RFD (≥ 100 ms) in trained women, but not men.

Only a few studies have compared the test–retest reliability across different standardized instructions. Similar intra‐session reliability for RFD across all time intervals (0–50 to 0–200 ms) was observed for the ankle and knee extensors when participants contracted “as hard and fast as possible” (HF) compared with brief, ballistic contractions performed as fast as possible, a protocol comparable to the present FO protocol [12]. In contrast, Jaafar & Lajili [10] reported significantly better test–retest reliability for knee extensor peak RFD as well as RFD derived between 25% and 50% of peak torque (T_25–50_%) when contracting as fast as possible (FO protocol) compared to HF. Likewise, Jaafar & Lajili [11] reported superior reliability for knee extensor peak RFD with FO compared to HF instruction.

In the present study, a clear pattern of numerically improved intersession reproducibility was observed for RTD and impulse with increasing duration of the time intervals regardless of instruction protocol and selection criteria, with lower reliability noted in early time intervals (0–30, 0–50 ms), as reflected by lower ICCs and higher CV_w‐s_, compared to the later time intervals (0–100, 0–200 ms). This trend is consistent with previous findings for isometric knee extensor RTD with both “Hard and fast” [5] and “fast” or ballistic instructions [6, 12, 30].

### 4.3 Isometric Peak Torque

In the present study, HF and HO instruction protocols were specifically compared to evaluate the effect of dual‐focus instruction (HF) versus solely aiming to reach maximal torque production by demanding a slow and gradual force production (HO). The results obtained demonstrate a minimal and statistically nonsignificant influence of verbal instruction on peak torque production across all test sessions. Previous research has produced somewhat conflicting findings, as some studies investigating handgrip [13, 14] and various isometric hand, elbow, and ankle strength tests [27] have reported instruction‐dependent differences in maximal isometric force. In contrast, others investigating isometric dorsiflexion tasks [28] and isometric mid‐thigh pull [29] have found no significant effect of instruction on isometric peak force. Collectively, these mixed observations suggest that the influence of verbal instruction on maximal isometric muscle strength may depend on the specific muscle group examined, the testing modality employed, and the test population examined.

### 4.4 Strengths and Limitations

In line with previous reports [12, 30], we opted to perform more trials interspaced by shorter rest intervals with FO instruction (10 trials, 30 s), compared to HF and HO (5 trials, 120 s), respectively. Unlike HF and HO, the brief contractions performed with FO without reaching maximal force production were expected to prevent the build‐up of muscular fatigue, thereby allowing a higher number of FO trials to be performed. However, as different numbers of trials were performed for HF and HO (five in each) versus FO (ten), it cannot be excluded that the different numbers of test contractions may have influenced the observed differences in rapid force production performance as well as test–retest reliability for rapid force production observed between the HF and FO instruction protocols.

To facilitate maximal performance, verbal encouragement and online visual (PC screen) feedback were both provided during and between trials. It is possible that participants might have been aware of their peak torque measurements from previous test sessions, which in turn could have influenced their motivation and hence affected their performance. Within each test session, participants were encouraged to continuously exceed their current best peak torque performance.

As a methodological strength of the present study, comprehensive intra‐rater intersession test–retest reliability data were obtained for three different instruction protocols (HO, HF, FO) combined with three different selection criteria (peak torque, maximal impulse, composite time intervals) across three separate test sessions. Such multifactorial designs are rare and offer a robust and practically relevant assessment of how testing and analytical approaches may influence test performance and measurement reliability.

## 5 Conclusion

In conclusion, when testing maximal isometric knee extensor strength and rapid force capacity (explosive strength) the choice of trial selection criterion and verbal instruction markedly influenced performance output and intersession test–retest reliability. Specifically, selection criteria based on maximal impulse produced in 0–200 ms (Max_Impulse200) and maximal composite RTD/impulse values (Max_Composite_Interval) both demonstrated improved test–retest reliability compared to the peak torque criterion (Max_Peak_Torque). Furthermore, higher RTD/impulse values were recorded, and measures of test–retest reliability were numerically improved when participants were instructed to contract as fast as possible (FO) compared to as hard and fast as possible (HF). These instruction‐specific differences (HF vs. FO) in test–retest reliability estimates were observed to attenuate between test sessions 2–3 compared to test sessions 1–2. Conversely, instruction‐specific differences in RTD/impulse outcome values were observed to increase in the latter test sessions. Taken together, these findings imply that test familiarization can reduce test variability with both instructions (HF and FO), thereby leading to improved intersession reliability (manifested by higher ICCs and lower CV_w‐s_ and SDD). Notably, measurement of knee extensor peak torque strength remained highly reliable across repeated test sessions, irrespective of verbal instruction (HO vs. HF).

## 6 Perspectives

The present study adds methodological insight into maximal and rapid knee extensor strength assessments. By showing that different verbal instruction protocols and trial selection criteria notably affect both performance outcomes and test–retest reliability, the present findings offer practical guidance toward more standardized and reliable testing procedures. Across instruction and selection criteria, intersession reliability for RTD and impulse improved between later test sessions, indicating a familiarization session should be considered when assessing rapid force production. Employing a “Fast Only” instruction resulted in increased indices of rapid knee extensor force production while concurrently providing stable and reproducible measurements, whereas peak torque values remained unaltered by verbal instruction, while concurrently demonstrating excellent reliability. Accordingly, FO instruction may be preferred when assessing maximal RTD and impulse, while the choice of instruction appears less critical for peak torque assessment. The present impulse‐based selection criterion (Max_Impulse200) yielded more reliable measures of rapid force production compared to the commonly used peak torque‐based criterion (Max_Peak_Torque) during Hard and Fast verbal instruction, with only very minor further improvements in reliability observed when using a composite selection approach. Future research should examine whether these methodological recommendations translate to other test modalities, clinical populations, and sport‐specific performance assessments.

## Funding

The authors have nothing to report.

## Conflicts of Interest

The authors declare no conflicts of interest.

## Data Availability

The data that support the findings of this study are available on request from the corresponding author. The data are not publicly available due to privacy or ethical restrictions.
